# Desmoid fibromatosis infiltrating left adrenal gland and kidney

**DOI:** 10.1093/jscr/rjac585

**Published:** 2022-12-25

**Authors:** Sui Wu Tee, Avisha Richards, Yi Han Tan, Dhinisya Jeyabalan, Gunaseelan Durairaj

**Affiliations:** Department of General and Vascular Surgery, Serdang Hospital, Selangor, 43000 Kajang, Malaysia; Division of Clinical and Surgical Sciences, University of Edinburgh, Edinburgh EH8 9YL, UK; Department of General and Vascular Surgery, Serdang Hospital, Selangor, 43000 Kajang, Malaysia; Department of General and Vascular Surgery, Serdang Hospital, Selangor, 43000 Kajang, Malaysia; Department of General and Vascular Surgery, Serdang Hospital, Selangor, 43000 Kajang, Malaysia; Department of General and Vascular Surgery, Serdang Hospital, Selangor, 43000 Kajang, Malaysia

## Abstract

Desmoid fibromatosis is a rare, benign, locally aggressive fibroblastic proliferation that may occur in almost any anatomical location. Due to its rarity and unpredictable clinical course, there has not been a standard guideline of treatment. We encountered a case of desmoid fibromatosis in our centre. A young lady previously fit and well was referred for a symptomatic, rapidly growing left sided abdominal mass. Otherwise, she denied any bowel related symptoms or constitutional manifestation. Imaging demonstrated a large well-defined lobulated solid-cystic mass extending from vertebral level T10 to L5, measuring 10.5 cm × 15 cm × 23 cm. The mass was in close proximity with the left adrenal gland, left kidney, pancreas and spleen. Ultrasound guided biopsy interpreted it as a fibroblastic or myelofibroblastic tumour, favouring desmoid fibromatosis. Surgery was then performed where the mass was removed along with the left adrenal gland and kidney. Post-operative care was complicated with pulmonary embolism, hospital-acquired pneumonia and pancreatitis.

## INTRODUCTION

Desmoid tumour, also known as aggressive fibromatosis, is a type of benign but locally aggressive tumour that arises from fascial or musculoaponeurotic structure. As such, the tumour may develop anywhere in the human body, be it intrabdominal or extra-abdominal. These tumours are rare, with an estimated incidence of 2–4 per million per year, and they only account for 0.03% of all neoplasm. Even though these tumours exhibit negligible metastatic potential, they remain difficult to treat as they tend to enlarge rapidly and cause pressure effect or obstruction to its surrounding structures, which can be fatal [1]. Due to the rarity of these tumours, there are limited data and studies on them.

## CASE PRESENTATION

We encountered a case of desmoid tumour in our centre. A young lady was referred to the outpatient clinic with a painless abdominal mass. It was progressively increasing in size over the past 3 months, associated with discomfort. She denied any altered bowel habit, previous trauma, urinary and constitutional symptoms. On examination, the abdomen was soft with a 10 cm × 15 cm mass palpable over the left flank, firm in consistency. There was no lymphadenopathy palpable.

**Figure 1:**
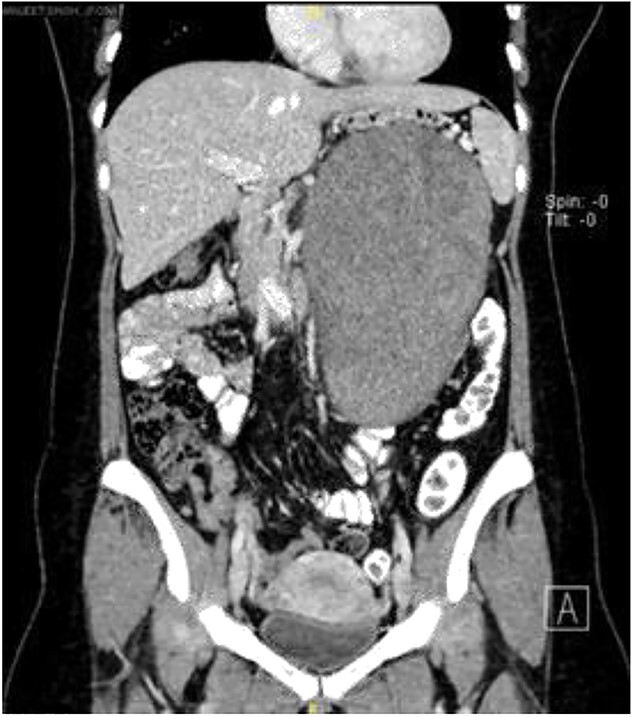
Coronal view of computerized tomography abdomen

Computerized tomography scan demonstrated a large, well-defined lobulated heterogeneously enhancing solid cystic mass sized 10.7 cm × 12.7 cm × 22.2 cm. The mass displaced the pancreas anteriorly, and it compressed onto the splenic vein with collateral vessels seen, draining into the portal vein. Posteriorly, the lesion displaced the left kidney posteriorly and compressed onto the left adrenal gland superiorly. The remaining structures were unaffected (Fig. 1).

**Figure 2:**
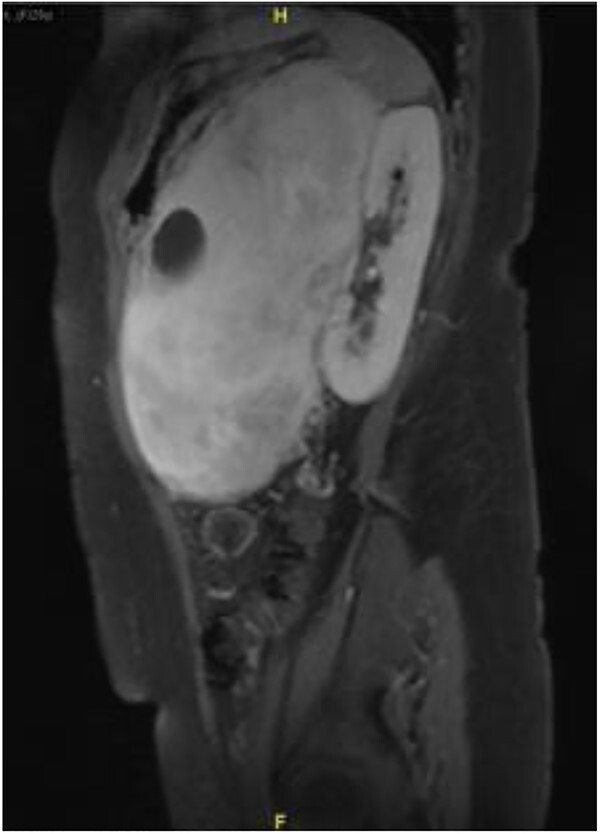
Sagittal view on magnetic resonance imaging abdomen

Subsequently, a magnetic resonance imaging was performed to better delineate fat plane with adjacent organs. Similarly, it showed a large left retroperitoneal solid-cytic mass with mass effect and poor fat plane between it and the left kidney, left adrenal gland, splenic hilum and pancreas. Additionally, posteromedially, the mass appeared to be abutting the abdominal aorta at L2 level with poor fat plane in between. Fortunately, no obvious invasion or filling defect was noted within (Fig. 2).

**Figure 3:**
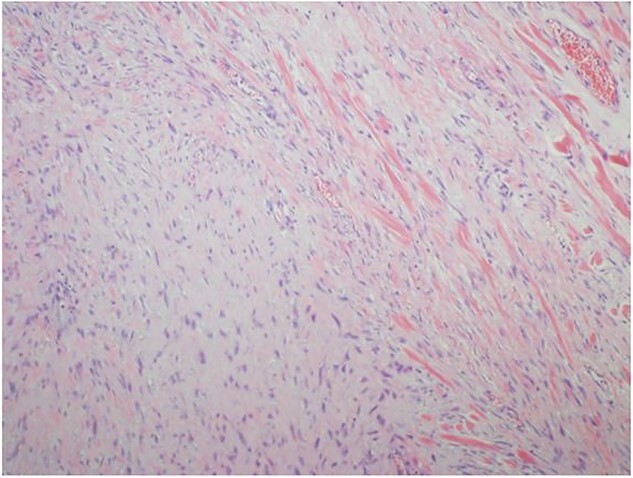
Histopathological findings. Spindle cells fascicles with collagen formation (H&E ×100).

**Figure 4:**
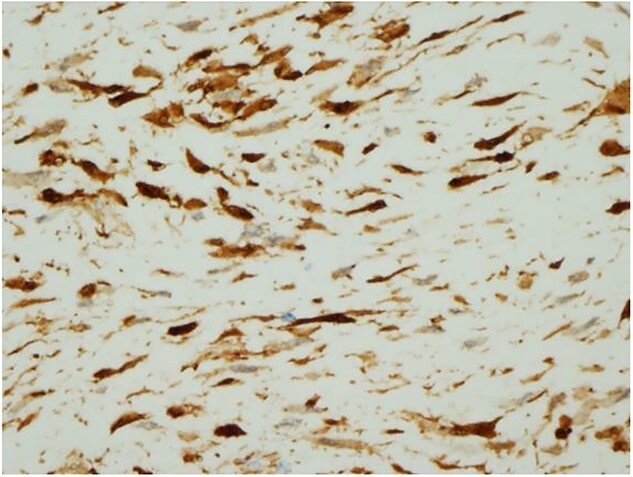
Histopathological findings. The tumour cells are positive for beta-catenin (H&E ×400).

Ultrasound guided biopsy was performed, which showed spindle cells fascicles with collagen formation. The cells were stained positive to beta-catenin nuclear staining, and negative for SMA, CD34, Desmin and S100 (Figs 3 and 4). The diagnosis of desmoid fibromatosis was confirmed.

Subsequent clinic visits demonstrated worsening of compressive symptoms, and Multidisciplinary Team meeting outcome was to list patients for elective excision of tumour, splenectomy, left nephrectomy and left adrenalectomy.

**Figure 5:**
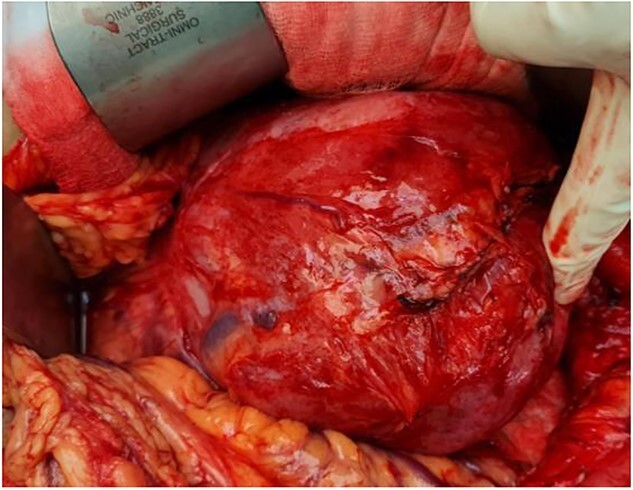
Intra-operatively, the tumour was stretching the mesocolon, and laterally adherent to the left kidney

**Figure 6:**
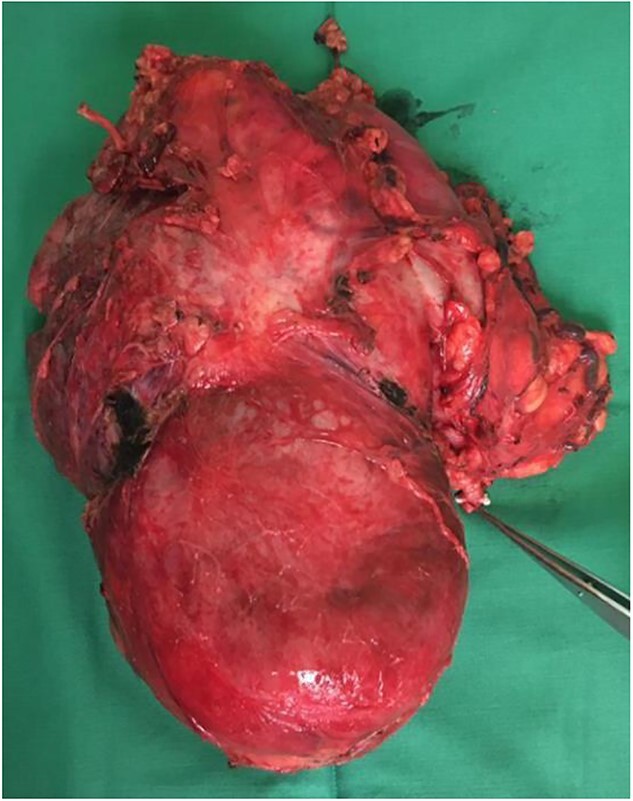
Tumour removed. Pointer demonstrates the left ureter.

Our initial approach was a midline laparotomy. Upon entering the peritoneum, the huge tumour sized 12 cm × 15 cm was identified. Inferiorly, the overlying transverse mesocolon was noted to be stretched. Laterally, the tumour was densely adherent to the medial aspect of the left kidney, and the left adrenal gland was not visualized due to the extensive involvement of the left kidney (Figs 5 and 6). Posteriorly, the tumour was also adhered to the duodenojejunal junction, spleen and pancreatic tail; however, we managed to shave off and preserve the organs. Intra-operatively, multiple units of transfusion were required. The tumour weighed 1.2 kg.

This patient’s post-operative care was complicated with hospital acquired pneumonia, pancreatitis and pulmonary embolism, requiring her to be placed in intensive care unit for 2 weeks. She subsequently recovered and was discharged well.

## DISCUSSIONS

Desmoid tumour is a rare tumour that exhibits fibroblastic proliferation that originates from fascial or musculoaponeurotic structure. Macroscopically, it has the appearance of a hard fibrous lump with pale-tan colour due to its poor vascularization. Microscopically, it contains dense collagenous material with interspersed spindle cells and fibroblast that has an abundant eosinophilic cytoplasm. The molecular pathogenesis of desmoid tumours is poorly understood, but APC gene mutations and beta-catenin accumulation are described in cases of desmoids with underlying Familial Adenomatous Polyposis (FAP).

Although the aetiology of these tumours is not well defined, it has been found to be associated with certain familial syndromes such as FAP and Gardner Syndromes. It occurs in 10–20% of patients diagnosed with FAP, but the risk of developing FAP in patients with desmoid fibromatosis is only 4–5%. Post-operatively, our patient was further investigated via endoscopy, and the results were negative.

Desmoid tumours are more common in females. 80% of desmoid tumours are found in females, and 50% occur in females of reproductive age, ranging from 20 to 40 years old. In view of its predominance in young women of reproductive age, it was proposed that environments with high oestrogen may promote its growth.

Due to the rarity and unpredictable behaviour of desmoid tumours, there has not been a standard of care, and individualized treatment by experts is required [2]. Treatment options vary and can be divided into conservative, surgical resection, radiotherapy, chemotherapy and hormonal treatment.

Currently, the ‘Wait and See’ approach is the recommended approach to desmoid fibromatosis, especially in stable and asymptomatic patients [3]. Data suggest that progression of disease is rare. The average time to progression is 14 months, and only 9% of the cases progressed after a 5-year interval. Hence, with close monitoring, if the tumour is stable over a period of 2 years, the risk of them experiencing progression later on is low [4].

Surgery as the first line of treatment has reduced in popularity due to its high risk of recurrence. It has been suggested that the risk of recurrence may be as high as 60% within 5 years [3, 4]. In addition, many tumours are located in unfavourable location, making surgical resection difficult. In the case of a large tumour, as in this case, there is significant morbidity associated, and to achieve R0 resection may be challenging. There is also a significant risk of bleeding in desmoid tumours surgeries. One of the case reports published by Georgiades et al demonstrated a patient with a small desmoid tumour presented as haemorrhagic shock [5]. Similarly, in this case, the patient required a large amount of blood products intra-operatively. Results from research also shows that event-free survival between patients undergoing surgery and conservative management did not differ [3]. If surgery is the method of choice, surgical resection margin should be cleared, as a positive or close (<1mm) resection margin acts as an independent prognostic factor to local recurrence [6].

Radiotherapy is another method of treatment in desmoid tumours. It is usually used as an adjuvant therapy for post-surgical patients, particularly in cases of incomplete resection [7]. Studies have shown that post-operative radiotherapy significantly improved the progression free survival as compared with surgery alone [8]. Radiotherapy is also beneficial in recurrent disease patients, with a 5-year local control rate of 67% [9].

There are ongoing trials with chemotherapy medications in the treatment of desmoid fibromatosis. Methotrexate-vinblastine was the only chemotherapy regime assessed in clinical trial setting until the DESMOPAZ trial, which compares the progression of disease between pazopanib and the methotrexate-vinblastine group. Pazopanib is an oral antoangiogenic agent targeting VEGF receptors, platelet-derived growth factor receptor-like protein and tyrosine kinases, which has shown to limit the progression of disease more successfully than the methotrexate-vinblastine group [10].

Hormonal treatment, in particular tamoxifen, has been used as the first line of systemic treatment in non-resectable desmoid tumours. Immunohistochemical studies from these tumours illustrate the presence of oestrogen receptor beta in 90% of them. However, the response rate to tamoxifen has shown to be low and, so far, there has been no clear benefit to the symptom change, size or MRI signal change [11]. There has been an increase in popularity with toremifene, a triphenylethylene selective oestrogen receptor modulator. It has been suggested that it influences the transforming growth factor-beta and beta-catenin pathways, leading to growing arrest and a reduction in the size of tumour [12].

## CONFLICT OF INTEREST STATEMENT

None declared.

## FUNDING

None.
